# Psychomotor development and attention problems caused by a splicing variant of *CNKSR2*

**DOI:** 10.1186/s12920-020-00844-4

**Published:** 2020-12-09

**Authors:** Yi Zhang, Tingting Yu, Niu Li, Jiwen Wang, Jian Wang, Yihua Ge, Ruen Yao

**Affiliations:** 1 Department of Medical Genetics and Molecular Diagnostic Laboratory, Shanghai Children’s Medical Center, Shanghai Jiao Tong University School of Medicine, Shanghai, 200127 People’s Republic of China; 2 Department of Neurology, Shanghai Children’s Medical Center, Shanghai Jiao Tong University School of Medicine, Shanghai, 200127 People’s Republic of China; 3 Department of Orthopedics Pediatric, Shanghai Children’s Medical Center, Shanghai Jiao Tong University School of Medicine, Shanghai, 200127 People’s Republic of China

**Keywords:** CNKSR2, Neurodevelopmental disorder, Splicing variant, Whole exome sequencing, Attention deficit

## Abstract

**Background:**

Mutations in *CNKSR2* have been described in patients with neurodevelopmental disorders characterized by childhood epilepsy, language deficits, and attention problems. The encoded protein plays an important role in synaptic function.

**Case presentation:**

Whole-exome sequencing was applied to detect pathogenic variants in a patient with clinical symptoms of psychomotor development, attention deficit, poor logical thinking ability, and an introverted personality, but without epilepsy or any significant electroencephalogram changes. Genetic study revealed a splicing mutation (c.1904 + 1G > A) and RT-PCR revealed aberrant splicing of exon 16, leading to a reading-frame shift and a truncated protein in the PH domain.

**Conclusions:**

This is the first report of a splicing variant of *CNKSR2*, and the unique clinical features of this pedigree will help extend our understanding of the genetic and phenotypic spectra of *CNKSR2*-related disorders.

## Background

Neurodevelopmental disorders including intellectual disability, attention-deficit/hyperactivity disorder, and language deficits are extremely heterogeneous, both clinically and genetically. Underlying pathogenic variants have been identified in genes involved in different neurodevelopmental processes, including cell proliferation, neuron migration, synapse formation, and myelination [1].

*CNKSR2*—a gene encoding postsynaptic density proteins—plays an important role in neuronal proliferation, migration, differentiation, and death, as well as Ras-mediated synaptogenesis [2]. Impaired synaptic function caused by loss of *CNKSR2* has been indicated in patients with seizures, and intellectual, attention, and language deficits [3]. Herein, we report a patient with clinical symptoms including attention deficit, poor logical thinking ability, and an introverted personality (but without epilepsy or electroencephalogram changes) caused by an out-of-frame exon deletion due to a splicing variant of *CNKSR2*. This is the first reported case with a splice variant in *CNKSR2*, which could enhance our understanding of the genotypic and phenotypic spectra of *CNSKR2* in patients with neurodevelopmental disorders.

## Case presentation

The proband of the family was a six-year-old boy who sought help with attention deficit in school. He had an unremarkable prenatal history, with a birth weight of 2.95 kg and length of 50 cm at full term. The boy started walking alone at 18 months, and was diagnosed with a motor developmental delay in a local hospital. He started school at the normal age, but showed poor performance, especially in mathematics, and attention deficit in class. Language development was systematically evaluated as normal in the Department of Pediatric Developmental Behavior in Shanghai Children’s Medical Center using the Peabody picture vocabulary test-revised. The proband’s general cognitive ability, as estimated by the Wechsler Intelligence Scale for Children-Revised (WISC-R), is slightly below average indicating mild cognitive defects (Full scale IQ = 75). He had normal EEG and brain MRI results. He was raised by his grandparents and was described as very introverted in the presence of unfamiliar adults. The patient’s mother was a 33-year-old female with normal appearance. She worked in an office and completed her college education, although she exhibited poor performance in mathematics. The parents of the mother and father of the proband were all normal.

**Fig. 1:**
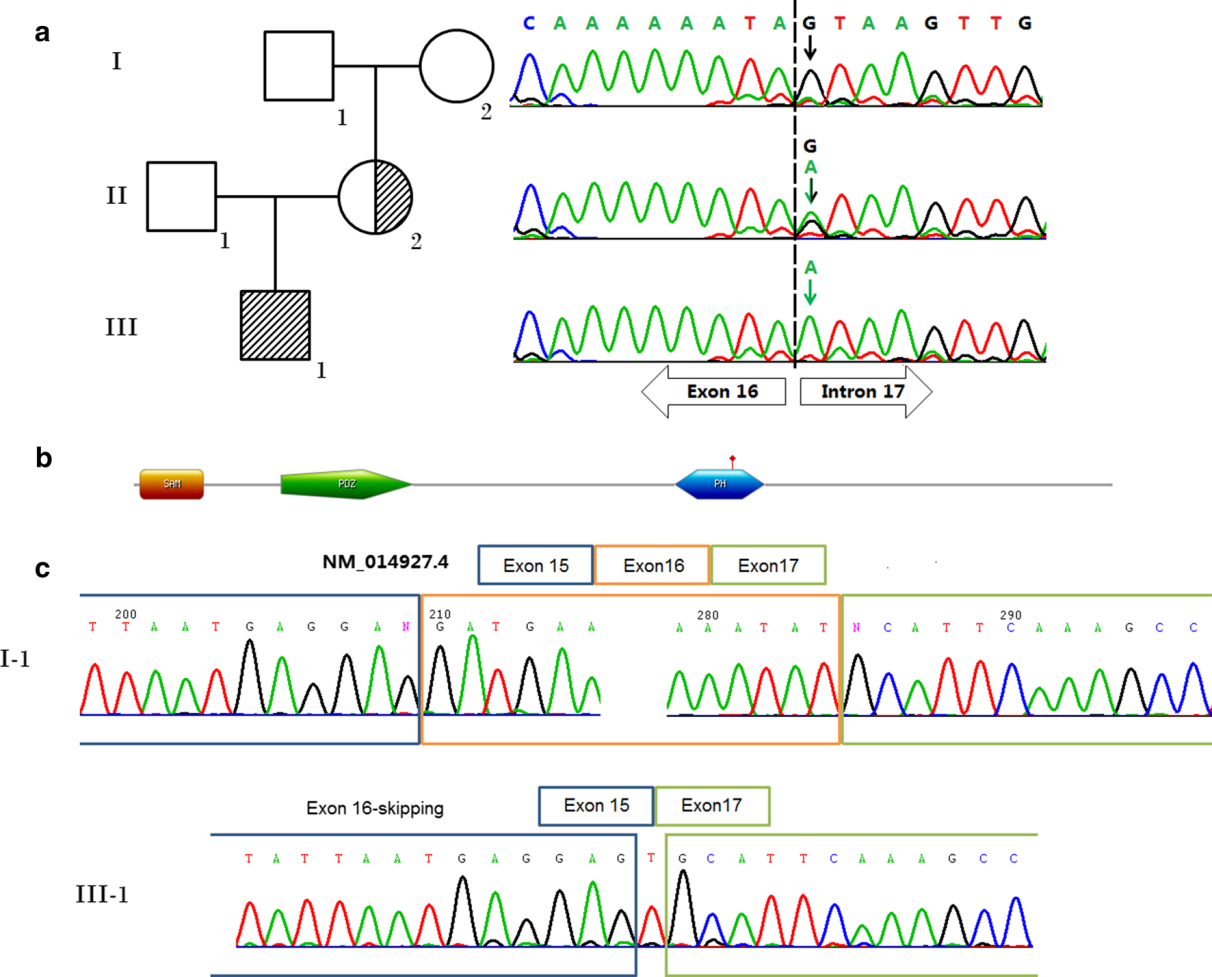
**a** Pedigree and Sanger sequencing confirmation of the splicing variant (c.1904 + 1G > A) of the *CNKSR2* gene. **b** Schematic map of variant location and domains of the *CNKSR2* gene. **c** Exon 16 skipping transcript detected from the patient caused by splicing variant

The patient’s peripheral blood DNA was subjected to WES to screen for causal variants. Details of WES was described in additional file (Additional file 1). A hemizygote splicing variant (c.1904 + 1G > A) of *CNKSR2* (NM_014927.4) in intron 17 (21 exons in total) was identified through WES in the patient and was considered as the possible disease-causing variant. Sanger sequencing was applied to confirm the variants (Fig. 1). The primers for amplification were designed using UCSC Exon Primer online software (http://genome.ucsc.edu/index.html) and synthesized. The primer sequences for the truncating variant to be confirmed were forward 5ʹ-TTACAGAGTATCATTACCTTCACACC-3ʹ and reverse 5ʹ-TGATTGACCTAGAAACTTCAGTGAC-3ʹ. Further pedigree investigation revealed the splicing variant was heterozygous in the mother, but wild type in both parents of the mother. According to the ACMG/AMP 2015 guidelines, the variant is categorized as pathogenic. As “G” in the position of *CNKSR2* 1904 + 1 is a consensus sequence at the splice-site, a mutation may induce abnormal splicing. Total RNA was extracted from peripheral blood of the patient and reverse transcription and subsequent PCR were performed to investigate alternative splicing products. RT-PCR was performed using primers spanning exons 13 through 20 (Fig. 1) in both the proband and control. The primer sequences were forward 5ʹ-caagacatcatgggcactcc-3ʹ and reverse 5ʹ-actccagtaatcttgttcctgc-3ʹ. Electrophoresis of the RT-PCR products did not show a significant difference in size. Direct sequencing analysis of the products revealed the induction of an exon 16-skipping product compared with the control, and the shift in reading-frame led to a termination of the protein after eight codons.

## Discussion and conclusions

Houge type of X-linked syndromic mental retardation is thought to be associated with hemizygous or heterozygous variants in *CNKSR2* on chromosome Xp22. Patients with pathogenic *CNKSR2* variants exhibit delayed development, major intellectual disability, speech and language delay, and early-onset seizures; continuous spike-wave activity or centrotemporal spikes are observed in EEGs. Since the first case of a patient lacking *CNKSR2*—due to a deletion of the initial 15 exons of the gene—was reported, seven more pedigrees with a similar phenotype and different *CNKSR2* variants have been reported [3–7]. These mutations include deletions covering all or part of the gene, frameshift-premature termination mutations, or stop-gain mutations, leading to loss-of-function of *CNKSR2*.

All previously reported male patients exhibited onset of seizures from neonatal stage to 3.5 years of age [3, 4] and most had characteristic frequent or continuous spike and wave EEG patterns. Language impairment was a cardinal feature of the reported patients; speech delay was noticed from onset of seizures and persisted indefinitely, leading to the absence of speech [3]. Developmental and behavioral challenges including severe attention deficit and hyperactivity were also recognized in patients. Other common and uncharacteristic features include intellectual disability and psychomotor delay (Table 1). There is only one female carrier in all reported literatures and exhibit with only mild learning disability or completely normal intellectual state.

**Table 1:** Comparison of reported pedigrees and cases with CNKSR2 gene variants

Pedigree no	Publication	CNKSR2 variant	Language defect	Attention problems	Seizure	Sleep EEG	Psychomotor delay	Female carrier
1 (two siblings)	Vaags et al.	arr[hg19]Xp22.12(20,297,696–21,471,387) × 0[mat]	Yes	Yes	Yes	Continuous spike-and-slow-waves	Yes	Mild learning disability
2	Vaags et al.	arr[hg19]Xp22.12(21,375,312–21,609,484) × 0[mat]	Yes	Yes	Yes	Continuous spike-and-slow-waves	Yes	N/A
3 (two siblings)	Vaags et al.	arr[hg19]Xp22.12(21,193,947–21,707,169) × 0[mat]	Yes	Yes	One sibling without seizure	No	Yes	N/A
4 (three siblings)	Vaags et al.	c.452insA p,D152Rfs*8	Yes	Yes	Yes	N/A	Yes	N/A
5 (three siblings)	Damiano et al.	c.2314 C > T; p.Arg712*	Yes	Yes	Yes	Centrotemporal or frontal spike and wave activity	Yes	Febrile seizures
6	Aypar et al.	arr[hg19]Xp22.12(21,328,677–21,670,497) × 0[mat]	Yes	N/A	Yes	Frequent and continuos centro-temporal spike and wave	Yes	Normal
7	Houge et al.	arr[hg19]Xp22.12(21,285,233–21,519,405) × 0[mat]	Yes	Yes	Yes	N/A	Yes	Normal
8	Sun et al	. c.2185C > T, p.Arg729*	Yes	Yes	Yes	Continuous spike-and-wave pattern	Yes	N/A
Our case		c.1904 + 1G > A	No	Yes	No	No	Mild	Mild learning disability

In the present case, the de novo occurrence of the splicing variant in the boy's mother strongly supports a causative role of this mutation. RT-PCR of the coding sequence of the gene confirmed out-of-frame deletion of exon 16 and a premature termination of *CNKSR2* in the PH domain. The patient and his mother showed very mild cognitive defects, which could be considered subclinical, such as attention deficit, poor logical thinking ability, and introverted personality.

Cases involving deletion of the entire *CNKSR2* or the N-terminal of the gene [3, 4] are thought to lead to loss of the CNK2. A frameshift mutation in the N-terminal of *CNKSR2* leads to an early stop-codon, possibly producing a non-functional protein product of 160 amino acids. The stop-codon variant detected in the 712 codon retained the SAM, PDZ, and PH domains of CNKSR2, but lacked the C-terminal of the gene. The out-of-frame deletion and reading-frame shift detected in our pedigree resulted in a truncated PH domain. The PH domain, located in the C-terminal of the gene, is known to stimulate the MAPK pathway [8] and both isoforms of CNK2 are located synaptically through the PH domain [9]. A possible reason for the relatively mild symptoms observed might be that the skewed PH domain of the truncated CNK2 protein gained a new function. As this variant is the first *CNKSR2* splicing variant detected, and aberrant splicing was confirmed from peripheral blood not from neuron tissues, it is possible that localized splicing and transcription of *CNKSR2* was different and maintained some level of functioning CNK2 protein.

One reason why *CNKSR2* variants are rarely detected may be that exonic deletions or small deletions encompassing *CNKSR2* are neglected by exome sequencing or targeted gene panel sequencing, which are widely used for identifying the genetic background of patients with neurodevelopmental disorders. Thus, evaluating exonic copy number variants with additional tests such as sensitive exon-level copy number arrays is worth considering to improve diagnostic efficiency.

In conclusion, a c.1904 + 1G > A variant in the *CNKSR2* is the first to be identified pathogenic splicing variant in patients, which broadens the spectrum of genetic variants of this gene.

## Supplementary information

**Additional file 1.** Details of whole exome sequencing. Whole exome sequencing details for pathogenic variants detection.

## Data Availability

The datasets (whole-exome sequencing and Sanger sequencing files) used and/or analysed during the current study are available in NCBI Sequence Read Archive (SRA), SRR13105481.

## Abbreviations

WES: Whole-exome sequencing
RT-PCR: Reverse transcription-polymerase chain reaction
EEG: Electroencephalogram
MRI: Magnetic resonance imaging

## References

[1] Wilfert AB, Sulovari A, Turner TN, Coe BP, Eichler EE (2017). Recurrent de novo mutations in neurodevelopmental disorders: properties and clinical implications. Genome Med.

[2] Lim J, Ritt DA, Zhou M, Morrison DK (2014). The CNK2 scaffold interacts with vilse and modulates Rac cycling during spine morphogenesis in hippocampal neurons. Curr Biol.

[3] Vaags AK, Bowdin S, Smith ML, Gilbert-Dussardier B, Brocke-Holmefjord KS, Sinopoli K (2014). Absent CNKSR2 causes seizures and intellectual, attention, and language deficits. Ann Neurol.

[4] Aypar U, Wirrell EC, Hoppman NL (2015). CNKSR2 deletions: a novel cause of X-linked intellectual disability and seizures. Am J Med Genet A.

[5] Damiano JA, Burgess R, Kivity S, Lerman-Sagie T, Afawi Z, Scheffer IE (2017). Frequency of CNKSR2 mutation in the X-linked epilepsy-aphasia spectrum. Epilepsia.

[6] Houge G, Rasmussen IH, Hovland R (2012). Loss-of-function CNKSR2 mutation is a likely cause of non-syndromic X-linked intellectual disability. Mol Syndromol.

[7] Sun Y, Liu YD, Xu ZF, Kong QX, Wang YL (2018). CNKSR2 mutation causes the X-linked epilepsy-aphasia syndrome: A case report and review of literature. World J Clin Cases.

[8] Therrien M, Wong AM, Kwan E, Rubin GM (1999). Functional analysis of CNK in RAS signaling. Proc Natl Acad Sci U S A.

[9] Yao I, Ohtsuka T, Kawabe H, Matsuura Y, Takai Y, Hata Y (2000). Association of membrane-associated guanylate kinase-interacting protein-1 with Raf-1. Biochem Biophys Res Commun.

