# Supplementary material for: Psychomotor development and attention problems caused by a splicing variant of CNKSR2
Source: BMC Med Genomics. 2020 Dec 9;13:182. doi: 10.1186/s12920-020-00844-4 (PMC7727132; doi:10.1186/s12920-020-00844-4)
Supplement: Supplementary file 1 — Additional file 1. Details of whole exome sequencing. Whole exome sequencing details for pathogenic variants detection. [file 12920_2020_844_MOESM1_ESM.docx]

Details of whole exome sequencing

Peripheral blood samples were collected from the patients and their parents. Medical exome sequencing was performed using Agilent SureSelect capture kits (Agilent, Santa Clara, California, United States). The captured libraries were sequenced using the Illumina HiSeq 2500 system, and the base calling and sequence read quality assessment were performed using Illumina HCS 2.2.58 software. Sequencing reads were aligned to hg19 using BWA-MEM and variant calling was performed with Genome Analysis Toolkit. All single nucleotide variants and indels were saved as VCF format files, and annotated with both Ingenuity® Variant Analysis™ (Ingenuity Systems, Redwood City, California, USA) and TGex (Translational Genomic Expert) for variation filtering and interpretation. All the variations exonic and splicing variants in genes with related disorders were classified according to the recommended method of the American College of Medical Genetics and Genomics (Richards et al., 2015). Pathogenic and potentially pathogenic variants were confirmed by Sanger sequencing and validated by parental testing.
